# The EQ-5D-5L is a valid approach to measure health related quality of life in patients undergoing bariatric surgery

**DOI:** 10.1371/journal.pone.0189190

**Published:** 2017-12-18

**Authors:** Jilles M. Fermont, Jane M. Blazeby, Chris A. Rogers, Sarah Wordsworth, on behalf of the By-Band-Sleeve Study Management Group

**Affiliations:** 1 Department of Medicine, Experimental Medicine and Immunotherapeutics, University of Cambridge, Cambridge, United Kingdom; 2 Department of Public Health & Primary Care, Cardiovascular Epidemiology Unit, University of Cambridge, Cambridge, United Kingdom; 3 School of Social and Community Medicine, Centre for Surgical Research, University of Bristol, Bristol, United Kingdom; 4 University Hospitals Bristol National Health Service Foundation Trust, Division of Surgery, Head and Neck, Bristol, United Kingdom; 5 School of Clinical Sciences, Clinical Trials and Evaluation Unit, University of Bristol, Bristol, United Kingdom; 6 Nuffield Department of Population Health, Health Economics Research Centre, University of Oxford, Oxford, United Kingdom; 7 Oxford National Institute for Health Research Biomedical Research Centre, University of Oxford, Oxford, United Kingdom; Medical University of Vienna, AUSTRIA

## Abstract

Bariatric surgery is considered an effective treatment for individuals with severe and complex obesity. Besides reducing weight and improving obesity related comorbidities such as diabetes, bariatric surgery could improve patients’ health-related quality of life. However, the frequently used instrument to measure quality of life, the EQ-5D has not been validated for use in bariatric surgery, which is a major limitation to its use in this clinical context. Our study undertook a psychometric validation of the 5 level EQ-5D (EQ-5D-5L) using clinical trial data to measure health-related quality of life in patients with severe and complex obesity undergoing bariatric surgery. Health-related quality of life was assessed at baseline (before randomisation) and six months later in 189 patients in a randomised controlled trial of bariatric surgery. Patients completed two generic health-related quality of life instruments, the EQ-5D-5L and SF-12, which were used together for the validation using data from all patients in the trial as the trial is ongoing. Psychometric analyses included construct and criterion validity and responsiveness to change. Of the 189 validation patients, 141 (75%) were female, the median age was 49 years old (range 23–70 years) and body mass index ranged from 33–70 kg/m^2^. For construct validity, there were significant improvements in the distribution of responses in all EQ-5D dimensions between baseline and 6 months after randomisation. For criterion validity, the highest degree of correlation was between the EQ-5D pain/discomfort and SF-12 bodily pain domain. For responsiveness the EQ-5D and SF-12 showed statistically significant improvements in health-related quality of life between baseline and 6 months after randomisation. The EQ-5D-5L is a valid generic measure for measuring health-related quality of life in bariatric surgery patients.

## Introduction

Obesity refers to a body mass index (BMI) of greater than or equal to 30 kg/m^2^ and increases the risk of morbidity and mortality from obesity-associated diseases and conditions including type 2 diabetes, osteoarthritis and cardiac disease [1, 2]. Individuals with a BMI ≥40 kg/m^2^or between 35 and 40 kg/m^2^ with comorbidities that could be improved by weight loss, are classified as having severe and complex obesity. With obesity rates expected to continue to rise in most countries, effective treatments for obesity are crucial. Standard obesity treatments include diet, exercise, behavioural interventions and drug therapy. However, for severe and complex obesity, bariatric surgery is now considered an effective treatment option and recommended by national bodies such as the National Institute for Health and Care Excellence (NICE) [3].

The most common types of bariatric surgery are laparoscopic Roux-en-Y gastric bypass, adjustable gastric band surgery and laparoscopic sleeve gastrectomy, with each having its respective benefits and risks. The Roux-en-Y gastric bypass restricts the volume of food eaten by creating a small thumb-sized pouch from the upper stomach and a bypass of the remaining stomach. Bypass alters physiology and anatomy in such a way as to achieve early and generally rapid weight loss but carries risks of serious early morbidity [4, 5]. Longer-term complications include the need for re-operation because of the development of internal hernias or intestinal obstruction and nutritional deficiencies. A gastric Band is an inflatable silicone device, which is placed around the top portion of the stomach to create a smaller stomach pouch, and weight loss is more gradual than with a Bypass. Short term surgical risks of an adjustable gastric band are low [5], but longer term complications include band erosion, migration or infection which may require revision surgery or band removal [4, 6]. Sleeve gastrectomy reduces the stomach to about 25% of its original size, removing a large portion of the stomach along the greater curvature, leaving a sleeve or tube-like structure. Complications include sleeve leakage resulting in a fistula and prolonged hospital stay, blood clots, infections, nausea and vomiting [7].

Many studies have suggested that bariatric surgery is effective at reducing not just weight loss and long-term morbidity, but also at improving health related quality of life (HRQOL) [8–14]. Generally, it takes several days or weeks to fully recover from surgery, depending on the type of surgery. However, it can take many months before patients are able to undertake activities that their weight had prevented them from achieving prior to surgery or even returning to their pre-surgery daily activities. Given the impact on general health, as well as the invasiveness of surgery, potential surgical complications and varied recovery time, HRQOL is clearly an important outcome for bariatric surgery.

A frequently used questionnaire to measure HRQOL is the EQ-5D. This is a generic health status questionnaire (i.e. it is not disease specific) and consists of a descriptive system and an EQ (EuroQol) visual analogue scale (VAS). Five dimensions are included in the descriptive system: mobility, self-care, usual activities, pain/discomfort and anxiety/depression. The latest version includes five levels in each dimension (EQ-5D-5L); from which respondents select the level which most closely matches their health state: no problems, slight problems, moderate problems, severe problems and extreme problems. The choices made within each domain relate to a 1-digit number and describe the respondent’s health state. Combining these digits results in a 5-digit number, which can be converted into a utility weight. The EQ-VAS is a 20 cm long vertical VAS where respondents can indicate their self-rated health ranging between the best and worst health states they can imagine, with zero representing death and 1 full-health. The EQ-5D has been used in a multitude of health conditions [15], has good test-retest reliability [16] and is validated for many diseases. However, despite its popularity and extensive use, to our knowledge, the EQ-5D has not been validated to measure HRQOL in patients undergoing bariatric surgery.

The aim of our study was to undertake a psychometric validation of the EQ-5D-5L to measure HRQOL in bariatric surgery patients. For the validation we used data from an on-going multi-centre randomised controlled trial (RCT) of alternative forms of bariatric surgery in the United Kingdom (UK) [17].

## Methods

### By-Band-Sleeve study

The By-band study gained National Health Service (NHS) ethics approval from the South West—Frenchay Research Ethics Committee (REC No: 11/SW/0248) on the 6th December 2011 and on the 8th May 2015 the Ethics Committee granted ethical approval to adapt the study from a two group (By-Band) to a three group (By-Band-Sleeve) trial. The REC approval applies to all NHS sites taking part in the study. The study is sponsored by the University of Bristol and it is the responsibility of the sponsor to ensure that all the conditions of the study are complied with. In addition, By-Band-Sleeve study was processed under pre-Health Research Authority (HRA) Approval systems, the study was granted HRA approval on the 24th July 2017.

The By-Band-Sleeve (BBS) study is a pragmatic three group RCT, as described in detail previously [17]. Initially the trial compared laparoscopic Roux-en-Y gastric bypass and adjustable gastric band surgery. A third group, laparoscopic sleeve gastrectomy was added after three years when it became apparent that this form of surgery was increasing in the UK [18, 19]. Here HRQOL data from the patients recruited earliest into the (Roux-en-Y gastric bypass and adjustable gastric band surgery) were used for the validation analyses. In addition, socio-demographic data collected in the BBS study were used.

### Study population

Adults with severe and complex obesity (BMI of ≥40 kg/m^2^, or a BMI of ≥35 kg/m^2^ with comorbidities) were eligible for the BBS study. Patients who were recruited between November 2012 (the start of recruitment to the trial) and March 2016 and who had reached their 6-month follow-up and had undergone surgery were included in the validation. Although the trial compares different types of surgery, because the study is on-going, information about participants’ allocation was not provided for any analysis. There are no guidelines on sample size requirements for instrument validation. However, a general recommendation is to have a minimum of 50–100 respondents [20].

### Psychometric validation

We used the Short Form-12 (SF-12), a subset of the SF-36, for the psychometric validation of the EQ-5D-5L as the SF-12 is being used within the BBS study. Wee *et al*. compared the SF-36 with the SF-12 in patients with and without obesity and concluded that the SF-12 was highly correlated with the SF-36 and superior to measure HRQOL differences related to BMI [21]. The SF-12 consists of 12 questions reflecting upon functional health and well-being. It includes eight domains: physical functioning, physical role limitations, bodily pain, general health, vitality, social functioning, emotional role limitations, mental health and two composite scores (a physical and mental component summary). Scoring is based on a 0 to 100 scale, where 100 represent the best HRQOL. Patients in the BBS study completed both the EQ-5D-5L and SF-12 at baseline (pre-randomisation) and at 6 months after randomisation.

Psychometric analyses for the validation were conducted according to the guidelines produced by the Scientific Advisory Committee of the Medical Outcomes Trust [22], and include construct and criterion validity, and responsiveness to change in health status over time.

*Construct validity* was assessed by examining the ability of the HRQOL instruments to discriminate between the health states of predefined groups over time. Groups that are expected to differ and are considered clinically relevant were predefined [23]. The following four groups were formed: (a) those with a BMI of <50 kg/m^2^ compared to those with a BMI of ≥50 kg/m^2^, and (b) those with any of and each of the following comorbidities (type I and/or type II diabetes, presence of obstructive sleep apnoea, New York Heart Association (NYHA) class II-IV, and unable to climb 3 flights of stairs) compared to those without. It was hypothesised that those with a BMI of ≥50 kg/m^2^ would have poorer HRQOL scores than those with a BMI of <50 kg/m^2^ [24–26], and those with comorbidity pre-surgery would have greater improvement in their HRQOL following surgery than those without comorbidity [10].

*Criterion validity* was assessed by examining the correlations between the domains of the different questionnaires, and by examining the correlations between the scores of the EQ-5D, EQ-VAS, and the SF-12 Physical (PHC) and Mental health composite (MHC) scores. Spearman’s correlation coefficients were calculated, with values <0.30 considered as negligible, 0.30–0.50 as moderate, and >0.50 as strong [27]. For the EQ-5D-5L version that is being used in the trial, UK EQ-5D-5L tariffs published by Devlin *et al*. were used [28].

*Responsiveness and sensitivity to change* were assessed by calculating (i) the HRQOL change scores (effect size (ES)), and (ii) the standardised response mean (SRM). These distribution-based methods are the two most widely used measures to assess the degree of observed change [29, 30]. While the ES (calculated by dividing the mean change in scores by the standard deviation (SD) at baseline) ignores the variation in change, SRM (calculated by dividing the mean change in scores by the SD of the change scores) makes change less sensitive to sample size because the SD of change is likely to be much smaller than the SD of the baseline scores, and is more similar to the paired *t-*test [31]. SRM scores are expected to be larger than ES scores, which is usual when assessing responsiveness and sensitivity to change in highly correlated variables, as SRM is a more efficient measure for observing change. An ES or SRM of 0.2 is considered small, 0.5 as medium, and 0.8 as large (Cohen’s thresholds).

### Data analysis

Socio-demographics are described using number and percentage for categorical variables such as diabetes, and the median (with interquartile range (IQR (Q_1_ –Q_3_))) for continuous variables such as age and BMI. Categorical data were compared using a Chi-square test, unless the expected cell frequency condition failed, in which case the Fisher’s exact test was used. Continuous data were compared using paired *t* tests if the distribution was approximately normal or the Wilcoxon matched-pairs signed-ranks test if the distribution was skewed.

Associations between the HRQOL instruments were quantified using Spearman’s rank correlations. It was expected that the correlation between the EQ-5D and SF-12 would be relatively high, as they are intended to measure very similar traits. The EQ-5D general health scores were compared between the predefined groups to assess construct validity (*t* tests). Categorical variables were coded as follows: diabetes (no diabetes *vs* any diabetes), BMI (<50 kg/m^2^ *vs* ≥50 kg/m^2^), NYHA (class I *vs* class II-IV), sleep apnoea (no apnoea *vs* apnoea), and functional status (3 flights of stairs *vs* <3 flights of stairs).

The change score (i) is expressed as Cohen’s ES and is the result of subtracting the mean HRQOL baseline score from the mean follow-up score (${\bar{x}}_{6\;\mathrm{months}}-{\bar{x}}_{\mathrm{baseline}}$), and dividing the mean change score by the SD of the baseline score. The SRM (ii) is the same mean change score divided by the SD of the change scores.

To determine if HRQOL change scores were of a minimal clinically important difference (MCID), patients in the validation sample were required to have achieved a certain level of weight reduction. Guidelines define 5–10% weight loss as a MCID [32–35]. However, relative weight reductions need to be larger to achieve MCIDs for some commonly used health status measures: 9% for EQ-5D Index, 23% for EQ-VAS, 23% for PHC (SF-36), and 25% for MHC (SF-36) scores [36]. For example, 9% weight loss would be expected to bring about a 0.03 improvement in the EQ-5D Index change score, the MCID for this instrument; relative weight loss <9% would not reflect a clinically important improvement in health status/utility measured with the EQ-5D Index. In contrast, the amount of weight loss has to be substantially greater (25%) in order to bring about a 5-point improvement in the SF-12 MHC, the MCID for this instrument. Individuals with a weight loss greater than or equal to the MCID weight cut-off point were considered improved. Individuals with a weight loss less than the MCID weight cut-off point were considered unchanged.

All tests were two-sided and of statistical significance at an alpha level of 0.05. Hypothesis testing to examine the construct validity of predetermined groups was one-sided. Our analyses were performed using Stata version 13 (College Station. Texas).

## Results

### Descriptive statistics socio-demographics

Complete health outcome data were available for 189 patients in the BBS study. Of the 189 patients included in the validation, 141 (75%) were female and the median age was 49 years old (range 23–70 years). The BMI of the cohort ranged from 33–70 kg/m^2^. The median weight was 131 kg and 65 patients (34%) had a BMI of ≥50 km/m^2^. Seventy-three patients (39%) had diabetes, 94% of whom were receiving medication such as oral hypoglycaemias. Forty-eight patients (25%) had obstructive sleep apnoea and most were receiving airway pressure treatment for the condition. Few of the patients (14%) had a diagnosis of cardiac disease (NYHA class II-IV). Hundred-two patients (54%) reported difficulty climbing one flight of stairs or less without resting (Table 1).

**Table 1:** Main patient characteristics at baseline and 6 months after randomisation (n = 189).

Characteristic	Number of patients (%) ^a^
Gender, female	141 (75)
Age (years), median (IQR)	49 (42–56)
Weight (kg) at baseline, median (IQR)	131 (116–148)
Weight (kg) at 6 months, median (IQR); missing	111 (98–128); 19 (10)
BMI (kg/m^2^) at baseline, median (IQR)	47 (42–52)
BMI (kg/m^2^) at baseline, ≥50 kg/m2	65 (34)
BMI (kg/m^2^) at 6 months, median (IQR)	40 (35–45)
BMI (kg/m^2^) at 6 months, ≥50 kg/m2; missing	28 (15); 19 (10)
Obstructive sleep apnoea at baseline	48 (25)
Obstructive sleep apnoea at 6 months; missing	39 (21); 21 (11)
Diabetes	73 (39)
NYHA class II-IV	27 (14)
Unable to climb ≥ 3 flights of stairs	102 (54)

IQR, interquartile range; BMI, body mass index; NYHA, New York Heart Association Functional Classification.

^a^ Number of patients and percentage given, unless otherwise indicated.

### Descriptive statistics health related quality of life data

**Fig 1:**
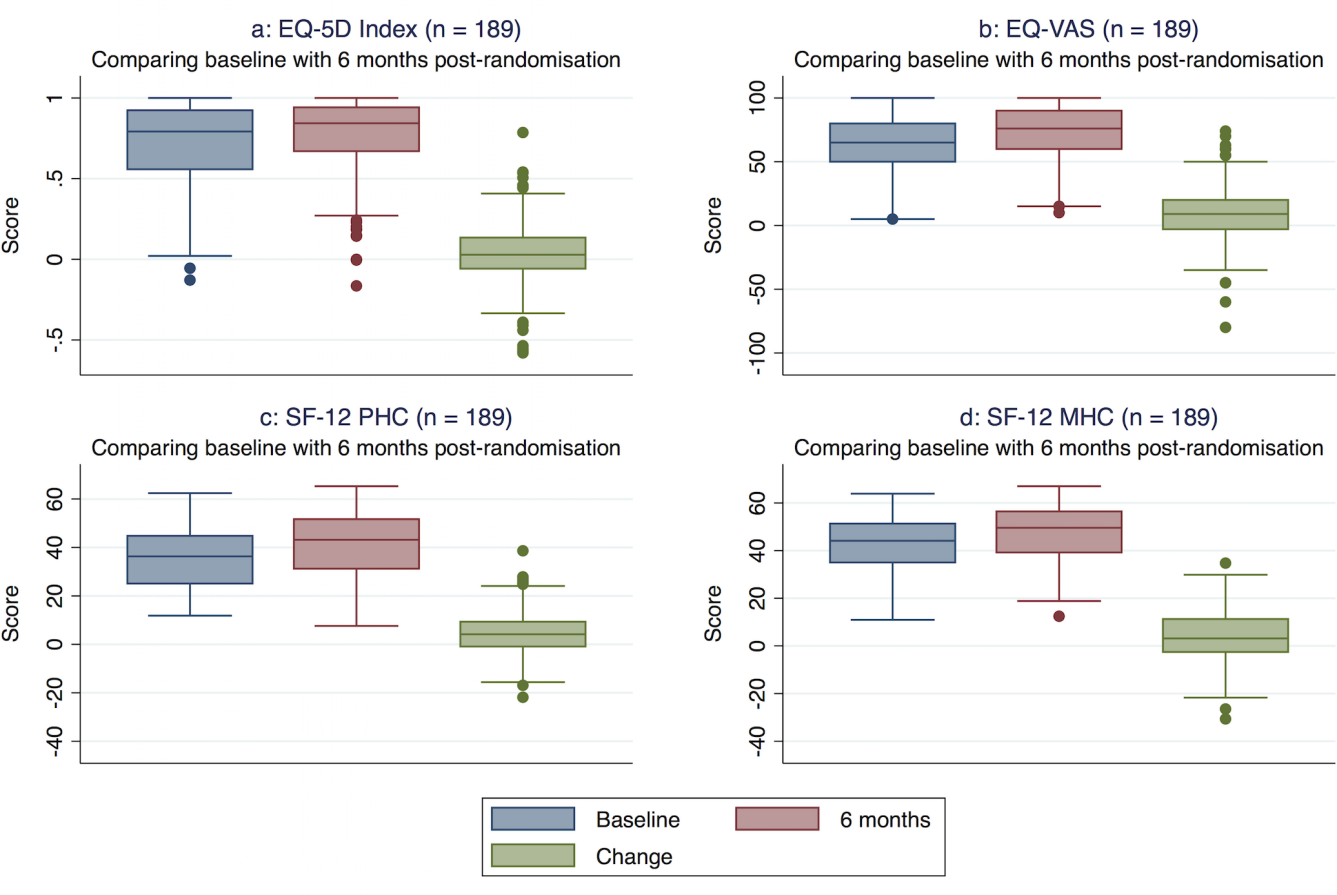
**a-d. Boxplots health realated quality of life scores at baseline and 6 months after randomisation including change scores (with quartiles and extreme scores) (n = 189).** VAS, Visual Analogue Scale; PHC, Physical Health Composite score; MHC, Mental Health Composite score.

Both the EQ-5D and SF-12 HRQOL scores improved from baseline to 6 months. The baseline average utility weight for the EQ-5D Index was 0.73 ± 0.25, which increased to 0.76 ± 0.25 6 months after randomisation (Table 2, Fig 1). Unlike the SF-12, the EQ-5D can be affected by a ceiling effect with a slightly higher proportion of patients reporting perfect health (maximum score) at 6 months after randomisation (21%) than at baseline (12%). The mean EQ-VAS score increased from 62 ± 21 at baseline to 71 ± 21 at 6 months after randomisation.

**Table 2:** Descriptive statistics and responsiveness of health related quality of life scores (n = 189).

Statistic	Mean ± SD	95% CI	Median (IQR)	Range	% Negative score	% Ceiling (maximum score)	*P* value ^a^
							(6 months after randomisation minus baseline)
**EQ-5D Index**							
Baseline	0.73 ± 0.25	0.69–0.76	0.79 (0.56–0.92)	-0.13–1	1	12	0.01
6 months	0.76 ± 0.25	0.73–0.80	0.84 (0.67–0.94)	-0.16–1	2	21	
Change	0.04 ± 0.20	0.01–0.70	0.03 (-0.06–0.13)	-0.58–0.79	-	-	
Cohen’s ES	0.16	-	-	-	-	-	
SRM	0.19	-	-	-	-	-	
**EQ-VAS**							
Baseline	62 ± 21	59–65	65 (50–80)	5–100	0	0	<0.01
6 months	71 ± 21	68–74	76 (60–90)	10–100	0	0	
Change	9 ± 22	6–12	9 (-3-20)	-80-74	-	-	
Cohen’s ES	0.44	-	-	-	-	-	
SRM	0.42	-	-	-	-	-	
**SF-12 PHC**							
Baseline	36 ± 12	34–37	36 (25–45)	12–62	0	0	<0.01
6 months	40 ± 13	38–42	43 (31–52)	8–65	0	0	
Change	4 ± 9	3–6	4 (0–9	-22-39	-	-	
Cohen’s ES	0.37	-	-	-	-	-	
SRM	0.47	-	-	-	-	-	
**SF-12 MHC**							
Baseline	43 ± 11	42–45	44 (35–51)	11–64	0	0	<0.01
6 months	47 ± 12	46–49	50 (39–56)	12–67	0	0	
Change	4 ± 12	2–6	3 (-3-11)	-31-35	-	-	
Cohen’s ES	0.38	-	-	-	-	-	
SRM	0.35	-	-	-	-	-	

SD, standard deviation; CI, confidence interval; VAS, Visual Analogue Scale; PHC, Physical Health Composite score; MHC, Mental Health Composite score; ES, Effect Size; SRM, Standardize Response Mean; Negative, scores below zero; Ceiling, ‘no problem’ answers to all questions. An effect size of 0.2 is considered small, 0.5 is medium and 0.8 is large.

^a^ *p* values were calculated using *t* tests.

### Construct validity

Between baseline and 6 months after randomisation, there were significant improvements in the distribution of responses in all the EQ-5D dimensions (Fisher’s exact test *P* <0.01; S1 Table).

We had hypothesised that patients with a BMI of ≥50 kg/m^2^ would have poorer HRQOL scores than those with a BMI of <50 kg/m^2^ and those with a comorbidity would have greater improvement in their HRQOL than those without. The EQ-VAS and the SF12-PHC were able to discriminate by BMI (<50 kg/m^2^ *vs* BMI ≥50 kg/m^2^; *t* tests EQ-5D *P* = 0.23, EQ-VAS *P* = 0.03, SF-12 PHC *P* = 0.02, SF-12 MHC *P* = 0.64; S2 Table). When assessing HRQOL change scores by comorbidity (as previously defined) *vs* no comorbidity, neither questionnaire was able to discriminate between those with and without any comorbidities (*t* tests EQ-5D *P* = 0.52, EQ-VAS *P* = 0.74, SF-12 PHC *P* = 0.84, SF-12 MHC *P* = 0.26). Also when exploring individual comorbidities, neither questionnaire was able to discriminate by comorbidity.

### Criterion validity

The direction and degree of correlation between the EQ-5D domains and SF-12 were as expected, with the highest degree of correlation between the EQ-5D pain/discomfort and SF-12 bodily pain domain *r* = -0.82 (Table 3). The negative direction of the coefficients can be explained by the fact that higher scores on the SF-12 represent better health, while higher scores on the EQ-5D represent worse health. Correlations of greater than or equal to 0.50 were considered as strong. For example, there was a strong negative correlation between EQ-5D mobility and SF-12 domains physical functioning *r* = -0.68. All correlations considered as strong are marked bold in Table 3.

**Table 3:** Correlations health related quality of life measures overall scores and domains, at baseline (n = 189).

	EQ-5D				
SF-12	Mobility	Self-care	Usual activities	Pain/ discomfort	Anxiety/ depression
Physical functioning	**-0.68**	**-0.51**	**-0.68**	**-0.63**	-0.35
Physical role limitations	**-0.65**	-0.46	**-0.67**	**-0.61**	-0.28
Bodily pain	**-0.69**	**-0.51**	**-0.75**	**-0.82**	-0.26
General health	-0.40	-0.30	-0.43	-0.47	-0.15
Vitality	-0.42	-0.34	-0.44	-0.41	-0.36
Social functioning	-0.46	-0.36	-0.46	-0.48	-0.32
Emotional role limitations	-0.30	-0.19	-0.29	-0.34	-0.45
Mental health	-0.29	-0.17	-0.25	-0.29	-0.43
	EQ-5D Index 5L	EQ-VAS	SF-12 PHC	SF-12 MHC	
EQ-5D Index	1.00	-	-	-	-
EQ-VAS	**0.50**	1.00	-	-	-
SF-12 PHC	**0.75**	**0.55**	1.00	-	-
SF-12 MHC	0.30	0.31	0.04	1.00	-

VAS, Visual Analogue Scale; PHC, Physical Health Composite score; MHC, Mental Health Composite score. Correlation coefficients with a values <0.30 are considered negligible, 0.30–0.50 as moderate, and >0.50 as strong [27].

The direction and degree of correlation between the different HRQOL components, except for the SF-12 MHC, were strong (Table 3). This was expected, as the instruments aim to measure the same traits. The ED-5D Index is most strongly correlated with the SF-12 PHC (*r* = 0.75).

### Responsiveness and sensitivity to change

Between baseline and 6 months after randomisation, both HRQOL measures showed statistically significant improvements (*t* tests, EQ-5D *P* = 0.01, EQ-VAS *P* <0.01, SF-12 PHC *P* <0.01, SF-12 MHC *P* <0.01; Table 2; Fig 1A–1D). The SRM (0.19) and ES (0.16) for the differences between baseline and 6 months after randomisation indicates that the EQ-5D, with a very small effect size, might be sufficiently sensitive to measure change but will possibly do this poorly. The SRM (0.42) and ES (0.44) of the EQ-VAS, SF-12 PHC (SRM 0.47; ES 0.37) and SF-12 MHC (SRM 0.35; ES 0.38) are also considered as small (Table 2).

From baseline to 6 months after randomisation there was a mean reduction in bodyweight of 20 ± 14 kg (n = 170). For the EQ-5D Index, 121 out of 170 patients (71%) met the cut-off point of having lost enough weight (≥9%) to have achieved the MCID for the EQ-5D Index, and were considered improved. For the EQ-VAS and SF12-PHC, 40 out of 170 patients (24%) met the weight loss cut-off point of 23% and were considered improved. For the SF12-MHC, only 26 out of 170 patients (15%) met the weight loss cut-off point of 25% and were considered improved. Except for the EQ-5D Index, the improved group had slightly lower baseline HRQOL scores compared to the unchanged group, but the mean change scores were greater in the improved group than in the unchanged group (*t* tests, EQ-5D *P* = 0.02, EQ-VAS *P* 0.04, SF-12 PHC *P* <0.01, and SF-12 MHC *P* 0.01; Table 4).

**Table 4:** Mean health related quality of life and change scores by minimal clinically important difference (n = 170).

Scale	Outcome by MCID				*P* value ^a^
	Unchanged group	Change score	Improved group	Change score	
	Mean ± SD	Mean ± SD	Mean ± SD	Mean ± SD	
EQ-5D Index	0.70 ± 0.26 (n = 49)	-0.02 ± 0.22	0.73 ± 0.24 (n = 121)	0.06 ± 0.20	0.02
EQ-VAS	63 ± 19 (n = 130)	7 ± 22	60 ± 23 (n = 40)	15 ± 22	0.04
SF-12 PHC	36 ± 12 (n = 130)	4 ± 8	34 ± 13 (n = 40)	9 ± 12	<0.01
SF-12 MHC	44 ± 11 (n = 144)	3 ± 12	42 ± 11 (n = 26)	9 ± 11	0.01

MCID, minimal clinically important difference; HRQOL, Health Related Quality of Life; VAS, Visual Analogue Scale; PHC, Physical Health Composite score; MHC, Mental Health Composite score. A change in HRQOL was considered to be of MCID when weight reductions were at least 9% for EQ-5D Index, 23% for EQ-VAS, 23% for SF-12 PHC, and 25% for SF-12 MHC scores.

^a^ *p* values for change scores were calculated using *t* tests.

## Discussion

This study has validated the EQ-5D-5L questionnaire to measure HRQOL in patients undergoing bariatric surgery. The validation analyses showed changes between baseline and 6 months after randomisation. There were significant improvements in the distribution of responses in all EQ-5D dimensions after surgery and the EQ-5D domains were appropriately correlated with the SF-12, confirming criterion validity. The EQ-5D is, therefore, recommended as a generic measure of HRQOL to be used in all trials evaluating surgery for severe and complex obesity.

Previous studies that have used the EQ-5D (generally the 3 level version) to measure HRQOL in bariatric surgery patients have produced mixed findings [36–41]. Van Mastrigt *et al* conducted an economic evaluation comparing vertical banded gastroplasty and laparoscopic band surgery to treat severe obesity [37]. No difference in HRQOL measured using the EQ-5D was found between the two interventions and the authors suggested that the EQ-5D might lack sensitivity to detect differences in surgical outcomes. Date *et al* found that the EQ-5D scores in the self-care, pain/discomfort, and anxiety/depression domains improved significantly after gastric bypass [38]. Mar *et al* assessed changes in EQ-5D scores after bariatric surgery and found increased problems with higher BMI scores. However, results indicate that the EQ-5D and other HRQOL questionnaires do not predict changes in HRQOL well with weight reduction [40]. The study included 79 severe obese patients with a mean weight reduction of 49 kg after two years. Others have usefully summarised the HRQOL measures that have previously been used in bariatric surgery [41].

When exploring the correlations between the two instruments in our study, the SF-12 domains, in particular bodily pain, physical limitations, and physical functioning showed strong negative correlation with the EQ-5D mobility, self-care, usual activities and pain/discomfort domains (i.e. better EQ-5D scores were associated with more mobility and less pain). The correlations between the EQ-5D and SF-12 in our study were consistent with, and slightly stronger than, those reported in previous studies undertaken in e.g. irritable bowel syndrome and heart disease settings [42–44] (S3 Table).

Ribaric *et al* concluded that the EQ-5D is sensitive enough to measure a minimally important difference, in HRQOL in bariatric surgery, measured at 3 years after surgery [39]. In the study by Warkentin *et al* HRQOL improvements were greatest within the first 6 months after surgery [36].

Most of these studies have used the older, 3 level version of the EQ5D rather than the most recent 5 level version, which should arguably be more sensitive to smaller changes in quality of life. When subjecting change scores to minimum weight loss cut-off points, we have been able to demonstrate that the EQ-5D is able to measure a minimally important difference in HRQOL over a time period of 6 months, which might support the premise that the EQ-5D 5 level version is more sensitive than the 3 level version.

At baseline, the mean EQ-5D-5L utility weight (0.73 ± 0.26) in our study was very similar than observed in a reference sample of individuals with a BMI similar to the BBS study (BMI ranging between 30kg/m^2^ and ≥40 kg/m^2^, mean utility weight 0.70 ± 0.27) [24]. Six months after randomisation, the mean EQ-5D-5L utility weight (0.76 ± 0.25) in the BBS study was lower than observed in a reference sample of individuals with a BMI in the ideal range of 18.5–25 kg/m^2^ (mean utility weight 0.80 ± 0.22), although higher to that observed in a reference sample of obese individuals with a BMI ranging between 30 and <35kg/m^2^ (mean utility weight 0.70 ± 0.29).

In terms of the limitations of our study, a larger sample size might have been useful, although we met the recommended minimum sample size requirements for a validation sample [20]. Results indicate that the EQ-5D did not capture mental health well in the sample studied. This is an important limitation as a publication in January 2016 has emphasised the importance of measuring the mental health impact of bariatric surgery [45]. Future, and large enough studies, evaluating the impact of bariatric surgery, might analyse subgroups based on the type, presence and severity of a mental health condition, as the EQ-5D is able to discriminate between severities and changes over time [46]. This might improve the ability of the EQ-5D-5L to capture change in mental health status. Until then we recommend the use of the EQ-5D-5L in combination with a mental health specific instrument. Also, we have not been able to demonstrate the ability to discriminate between groups based on BMI.

It is recommended that the EQ-5D-5L be used in studies measuring HRQOL in patients undergoing bariatric surgery. However, further work should explore in more detail the association between HRQOL and obesity specific parameters.

## Supporting information

S1 Table Distribution of EQ-5D dimension responses at baseline and 6 months (n = 189). ^a^ *p* values were calculated using *Fisher’s exact test*.
(DOCX)

S2 Table Known-groups validity (n = 189). ^a^ *p* values were calculated using *t* tests.
(DOCX)

S3 Table Predicted correlations between EQ-5D and SF-12 domains. Predictions on the correlations between the EQ-5D-5L and SF-12 [42–44]. Correlation coefficients with values <0.30 were considered negligible, 0.30–0.50 as moderate, and >0.50 as strong [27].
(DOCX)
