# Supplementary material for: The EQ-5D-5L is a valid approach to measure health related quality of life in patients undergoing bariatric surgery
Source: PLoS One. 2017 Dec 18;12(12):e0189190. doi: 10.1371/journal.pone.0189190 (PMC5734736; doi:10.1371/journal.pone.0189190)
Supplement: S1 Table — a p values were calculated using Fisher’s exact test. (DOCX) [file pone.0189190.s001.docx]

| **S1 Table. Distribution of EQ-5D dimension responses at baseline and 6 months (n = 189).** | | | |
| --- | --- | --- | --- |
| Dimension | Baseline n (%) | 6 months after randomisation n (%) | *P* value ^a^  (6 months after randomisation minus baseline) |
| **Mobility** |  |  | <0.01 |
| No problems | 75 (40) | 93 (49) |  |
| Slight problems | 39 (21) | 41 (22) |  |
| Moderate problems | 46 (24) | 29 (15) |  |
| Severe problems | 27 (14) | 25 (13) |  |
| Unable to walk about | 2 (1) | 1 (1) |  |
| **Self-care** |  |  | <0.01 |
| No problems | 129 (68) | 143 (76) |  |
| Slight problems | 42 (22) | 25 (13) |  |
| Moderate problems | 14 (7) | 20 (11) |  |
| Severe problems | 4 (2) | 1 (1) |  |
| Unable to wash or dress | - | - |  |
| **Usual activities** |  |  | <0.01 |
| No problems | 76 (40) | 95 (50) |  |
| Slight problems | 57 (30) | 47 (25) |  |
| Moderate problems | 5 (19) | 26 (14) |  |
| Severe problems | 18 (10) | 19 (10) |  |
| Unable to do usual activities | 3 (2) | 2 (1) |  |
| **Pain/discomfort** |  |  | <0.01 |
| No pain or discomfort | 35 (19) | 57 (30) |  |
| Slight pain or discomfort | 65 (34) | 64 (34) |  |
| Moderate pain or discomfort | 47 (25) | 34 (18) |  |
| Severe pain or discomfort | 33 (17) | 31 (16) |  |
| Extreme pain or discomfort | 9 (5) | 3 (2) |  |
| **Anxiety/depression** |  |  | <0.01 |
| Not anxious or depressed | 90 (48) | 101 (53) |  |
| Slightly anxious or depressed | 62 (33) | 55 (29) |  |
| Moderately anxious or depressed | 33 (17) | 24 (13) |  |
| Severely anxious or depressed | 3 (2) | 6 (3) |  |
| Extremely anxious or depressed | 1 (1) | 3 (2) |  |
