# Supplementary material for: The EQ-5D-5L is a valid approach to measure health related quality of life in patients undergoing bariatric surgery
Source: PLoS One. 2017 Dec 18;12(12):e0189190. doi: 10.1371/journal.pone.0189190 (PMC5734736; doi:10.1371/journal.pone.0189190)
Supplement: S2 Table — a p values were calculated using t tests. (DOCX) [file pone.0189190.s002.docx]

**S2 Table. Known-groups validity (n = 189).**

|  | EQ-5D Index | EQ-VAS | SF-12 PHC | SF-12 MHC |
| --- | --- | --- | --- | --- |
| Baseline score - Mean ± SD | | | | |
| **BMI at baseline (kg/m^2^)** |  |  |  |  |
| <50 kg/m^2^ (n = 124) | 0.74 ± 0.25 | 64 ± 20 | 37 ± 12 | 43 ± 10 |
| ≥50 kg/m^2^ (n = 65) | 0.71 ± 0.25 | 58 ± 21 | 33 ± 12 | 44 ± 12 |
| *P ^a^* | 0.23 | 0.03 | 0.02 | 0.64 |
| Change score - Mean ± SD | | | | |
| **Any comorbidity** |  |  |  |  |
| No (n = 41) | 0.04 ± 0.20 | 7 ± 24 | 3 ± 8 | 5 ± 11 |
| Yes (n = 148) | 0.04 ± 0.20 | 10 ± 21 | 5 ± 10 | 4 ± 12 |
| *P ^a^* | 0.52 | 0.74 | 0.84 | 0.26 |
| **Diabetes** |  |  |  |  |
| No (n = 116) | 0.02 ± 0.21 | 8 ± 22 | 4 ± 9 | 3 ± 12 |
| Yes (n = 73) | 0.07 ± 0.18 | 11 ± 21 | 5 ± 10 | 5 ± 11 |
| *P ^a^* | 0.94 | 0.85 | 0.69 | 0.82 |
| **Apnoea** |  |  |  |  |
| No (n = 141) | 0.04 ± 0.21 | 8 ± 23 | 4 ± 10 | 4 ± 12 |
| Yes (n = 48) | 0.02 ± 0.19 | 13 ± 17 | 6 ± 8 | 4 ± 12 |
| *P ^a^* | 0.25 | 0.90 | 0.83 | 0.56 |
| **NYHA** |  |  |  |  |
| Class I (n = 162) | 0.04 ± 0.19 | 10 ± 21 | 5 ± 10 | 4 ± 12 |
| Class II-IV (n = 27) | 0.03 ± 0.25 | 5 ± 23 | 4 ± 8 | 2 ± 11 |
| *P ^a^* | 0.42 | 0.12 | 0.28 | 0.17 |
| **Functional status** |  |  |  |  |
| ≥3 flights of stairs (n = 87) | 0.04 ± 0.17 | 11 ± 19 | 5 ± 9 | 4 ± 11 |
| <3 flights of stairs (n = 102) | 0.04 ± 0.23 | 8 ± 23 | 4 ± 10 | 4 ± 12 |
| *P ^a^* | 0.40 | 0.18 | 0.45 | 0.38 |
