# Supplementary material for: The EQ-5D-5L is a valid approach to measure health related quality of life in patients undergoing bariatric surgery
Source: PLoS One. 2017 Dec 18;12(12):e0189190. doi: 10.1371/journal.pone.0189190 (PMC5734736; doi:10.1371/journal.pone.0189190)
Supplement: S3 Table — Predictions on the correlations between the EQ-5D-5L and SF-12 [42–44]. Correlation coefficients with values <0.30 were considered negligible, 0.30–0.50 as moderate, and >0.50 as strong [27]. (DOCX) [file pone.0189190.s003.docx]

| **S3 Table. Predicted correlations between EQ-5D and SF-12 domains.** | | | | | | | |
| --- | --- | --- | --- | --- | --- | --- | --- |
|  | EQ-VAS | EQ-5D Mobility | EQ-5D Self-care | EQ-5D-5L Usual activities | EQ-5D-5L Pain/ discomfort | EQ-5D-5L Anxiety/ depression | EQ-5D-5L Health state |
| Negligible correlation <0.30 |  | Mental health | Mental health |  | Emotional role limitation | Physical functioning |  |
|  |  | Emotional role limitation | Emotional role limitation |  |  | General health |  |
|  |  |  | General health |  |  |  |  |
|  |  |  | Physical role limitations |  |  |  |  |
|  |  |  | Vitality |  |  |  |  |
| Moderate correlation  0.30 - 0.50 | Emotional role limitation | Vitality | Social functioning | Emotional role limitation | Mental health | Social functioning | Physical role limitations |
|  | Mental health | Physical role limitations | Bodily pain | Mental health | Vitality | Emotional role limitation | Emotional role limitation |
|  |  | Bodily pain | Physical functioning | Vitality | Physical role limitations | Vitality | General health |
|  |  | Social functioning |  | Bodily pain | Social functioning | Bodily pain | Vitality |
|  |  | General health |  | Social functioning | General health | Physical role limitations |  |
|  |  |  |  | General health |  |  |  |
| Strong correlation  >0.50 | Social functioning | Physical functioning |  | Physical functioning | Physical functioning | Mental health | Physical functioning |
|  | Vitality |  |  | Physical role limitations | Bodily pain |  | Bodily pain |
|  | Physical functioning |  |  |  |  |  | Social functioning |
|  | Physical role limitations |  |  |  |  |  | Mental health |
|  | Bodily pain |  |  |  |  |  |  |
|  | General health |  |  |  |  |  |  |
